# A comparative analysis of viral matrix proteins using disorder predictors

**DOI:** 10.1186/1743-422X-5-126

**Published:** 2008-10-23

**Authors:** Gerard Kian-Meng Goh, A Keith Dunker, Vladimir N Uversky

**Affiliations:** 1 Center for Computational Biology and Bioinformatics, Indiana University School of Medicine, Indianapolis, IN 46202, USA; 2 Institute for Intrinsically Disordered Protein Research, Indiana University School of Medicine, Indianapolis, Indiana 46202, USA; 3 Institute for Biological Instrumentation, Russian Academy of Sciences, 142290 Pushchino, Moscow Region, Russia; 4 Institute of Molecular & Cell Biology, 138673, Singapore

## Abstract

**Background:**

A previous study (Goh G.K.-M., Dunker A.K., Uversky V.N. (2008) Protein intrinsic disorder toolbox for comparative analysis of viral proteins. *BMC Genomics*. **9 **(Suppl. 2), S4) revealed that HIV matrix protein p17 possesses especially high levels of predicted intrinsic disorder (PID). In this study, we analyzed the PID patterns in matrix proteins of viruses related and unrelated to HIV-1.

**Results:**

Both SIV_mac _and HIV-1 p17 proteins were predicted by PONDR VLXT to be highly disordered with subtle differences containing 50% and 60% disordered residues, respectively. SIV_mac _is very closely related to HIV-2. A specific region that is predicted to be disordered in HIV-1 is missing in SIV_mac_. The distributions of PID patterns seem to differ in SIV_mac _and HIV-1 p17 proteins. A high level of PID for the matrix does not seem to be mandatory for retroviruses, since Equine Infectious Anemia Virus (EIAV), an HIV cousin, has been predicted to have low PID level for the matrix; i.e. its matrix protein p15 contains only 21% PID residues. Surprisingly, the PID percentage and the pattern of predicted disorder distribution for p15 resemble those of the influenza matrix protein M1 (25%).

**Conclusion:**

Our data might have important implications in the search for HIV vaccines since disorder in the matrix protein might provide a mechanism for immune evasion.

## Background

The viral matrix protein underlies the envelope of a virion, representing essentially a link between the envelope and the nucleocapsid [1,2]. The functions of matrix proteins are usually multifaceted, and not completely understood [3-5]. They are however known to be involved in the viral assembly and stabilization of the lipid envelope [6]. Matrix proteins of different viral types are often structurally, functionally, and evolutionarily related [4]. For instance, the influenza M1 and HIV p17 proteins are known to be related and both have similar RNA and membrane binding domains [4].

Lentivirinae is among the genii of viruses that possess a matrix layer [7,8]. Viruses that belong to this genus include Human Immunodeficiency Virus (HIV), Simian Immunodeficiency Virus (SIV), and Equine Infectious Anemia Virus (EIAV). The viruses in this family have different characteristics [7,9,10]. This is especially so with respect to the onset of diseases such as AIDS, the viral loads and the success or failure in finding vaccines.

There are three known HIV viruses in the world today, HIV-0, HIV-1, and HIV-2 [8,10,11]. The latter two are of the most interest to our study. The HIV-1 is the predominant virus spreading around the globe. HIV-2, by contrast, is predominantly spread in certain parts of Africa, being found in about 10% of HIV cases in West Africa, and has recently been found to be spreading in some parts of India [8,11]. While the onset of AIDS usually occurs within an average of 6 years of virtually all HIV-1 infections, those infected with HIV-2 are allowed a much longer time before the AIDS symptoms appear, if at all [8,11,12]. As for SIV, a few strains such as SIV_cpz _are more closely related to HIV-1, whereas most of the others, especially SIV_sm_, and SIV_mac_, are closer to HIV-2 [10]. It should be noted that SIV does not usually cause AIDS among African non-human primates [13]. It does however cause AIDS among Asia monkeys [14].

Similar to HIV and SIV, EIAV is another retrovirus [8,9], which, however, spreads by insects, and the host targets are non-cd4 white blood cells such as macrophages and monocytes [8,15]. The disease caused by EIAV is not usually as fatal to its host as that of HIV and ~90% of infected equine recovers from an initial onset of symptoms [8]. While the search for vaccines for HIV continues to be difficult and elusive, effective vaccine for EIAV had been found 20 years ago in China [10,15,16]. A major difficulty facing the search for HIV vaccines is a puzzling problem of the inability of HIV protein-binding antibodies in eliciting effective broad immune response [17]. While the reason for this remains largely unknown [18], a finding of high levels of intrinsically disordered proteins at the surface, envelope or perhaps, matrix could provide a mechanism by which the HIV virus evades the immune response.

Therefore, these data clearly show that related viruses might affect their hosts differently, possessing variable virulence and different modes of interaction with their host's immune systems. A question then arises is whether some of the mentioned variability in the behavior can be reflected in some peculiar features of the corresponding viral proteins. This paper examines matrix proteins of several related viruses using computational tools such as intrinsic disorder predictors to search for the crucial differences in the levels and distributions of intrinsic disorder in the matrix proteins.

The concept of protein intrinsic disorder is used in this paper to investigate characteristics pertaining to the various viral matrix proteins. Intrinsically disordered proteins have been described by other names such as "intrinsically unstructured" [19,20], "natively unfolded" [21,22], and "natively disordered" [23] among others. Historically, the investigation of intrinsic disorder began with finding and characterizing several proteins-exceptions from the paradigm stating that unique rigid protein structure is an unavoidable prerequisite for the specific protein function. Although such counterexamples were periodically observed, it was not till the end of the last century when researchers started to pay significant attention to this phenomenon [24]. As a result, the last decade witnessed the real rise of unfoldomics, a new field of protein science dealing with the various aspects of IDPs. It is recognized now that many crucial biological functions are performed by proteins which lack ordered tertiary and/or secondary structure; i.e., by IDPs [19-21,23-31]. The fact that amino acid sequences/compositions of IDPs and ordered proteins are rather different was utilized to develop numerous disorder predictors, which became instrumental in the pursuit of a greater understanding of intrinsic disorder. Access to important information on many of these predictors is provided via the DisProt database [32]. In this paper, we utilized two members of the PONDR^® ^family of disorder predictors, VLXT and VL3 [33-38], to examine the matrix proteins of the various viruses especially those related to HIV. PONDR^® ^VL3 was chosen because of its high accuracy in the prediction of long disordered regions [36], whereas PONDR^® ^VLXT was shown to be extremely sensitive for finding function-related disordered regions [35,39,40]. Uniqueness of this study is in the fact that we applied disorder predictors to proteins with known 3D-structure. This approach revealed some peculiar patterns of PID that can be used to better understand behavior of the HIV matrix proteins.

## Results

### Quantifying Disorder by Calculating the Percentage of Predicted Disordered Residues

**Figure 1:**
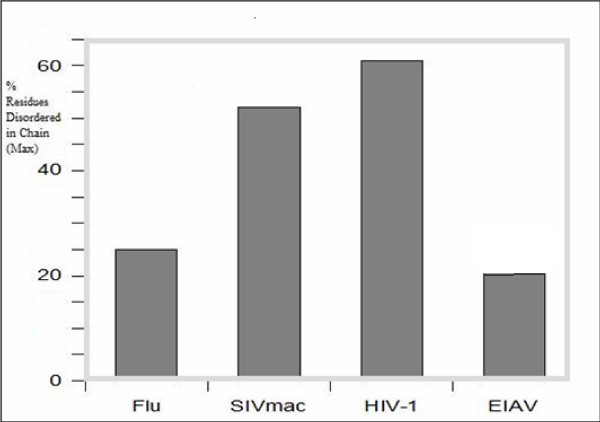
A bar chart comparing matrix proteins across virus types.

Table 1 represents the estimations of the percentage of predicted disordered residues in the analyzed matrix and capsid proteins. Even though influenza virus is quite unrelated to lentiviruses, its M1 matrix protein is placed here for comparison. It is important to remember also that the M1 protein is believed to be evolutionarily and structurally related to the p17 matrix protein of HIV. Table 1 shows that the amount of intrinsic disorder varies from 20 to 61%, and from 0 to 40%, being evaluated by PONDR^® ^VLXT and VL3 respectively. Data for the four matrix proteins with known 3-D structures are further illustrated by Figure 1 showing the results of the PONDR^® ^VLXT analysis as bar chart. High level of predicted intrinsic disorder in SIV and HIV-1 matrix proteins is clearly seen. In our earlier paper [41], the following classification of proteins characterized by X-ray crystallography but possessing various levels of predicted disorder was introduced: proteins with percentage of residues predicted to be disordered by PONDR^® ^VLXT between 20–29% were considered moderately disordered; those in the range of 30–39% were considered as quite disordered by prediction; whereas, proteins that were disordered 40% and above were considered as very disordered by prediction. Therefore, the influenza M1 protein and EIAV p15 should be considered as moderately disordered by prediction. By the same rule, the SIV_mac _and HIV-1 p17 matrix proteins have to be considered as highly disordered by prediction.

**Table 1:** A summary of matrix proteins and their percentages of disordered residues in a chain.

**Protein**	**Virus**	**Accession**	**% Predicted Disorder**
M1	Influenza A	1ea3	25 (0)
Matrix (p17)	SIV_mac_	1ed1	52 (40)
Matrix (p17)	HIV-1	1hiw	61 (39)
Matrix(p15)	EIAV	1hek	21 (12)
Capsid	HIV-1	1afv	48 (0)
Capsid	EIAV	1eia	30 (12)

All of the samples analyzed were structurally characterized using X-ray crystallography. Percentage of predicted disorder corresponds to values produced by the PONDR^® ^VLXT (VL3) analyses

### PONDR/B-Factor Plots and Contact Points

**Figure 2:**
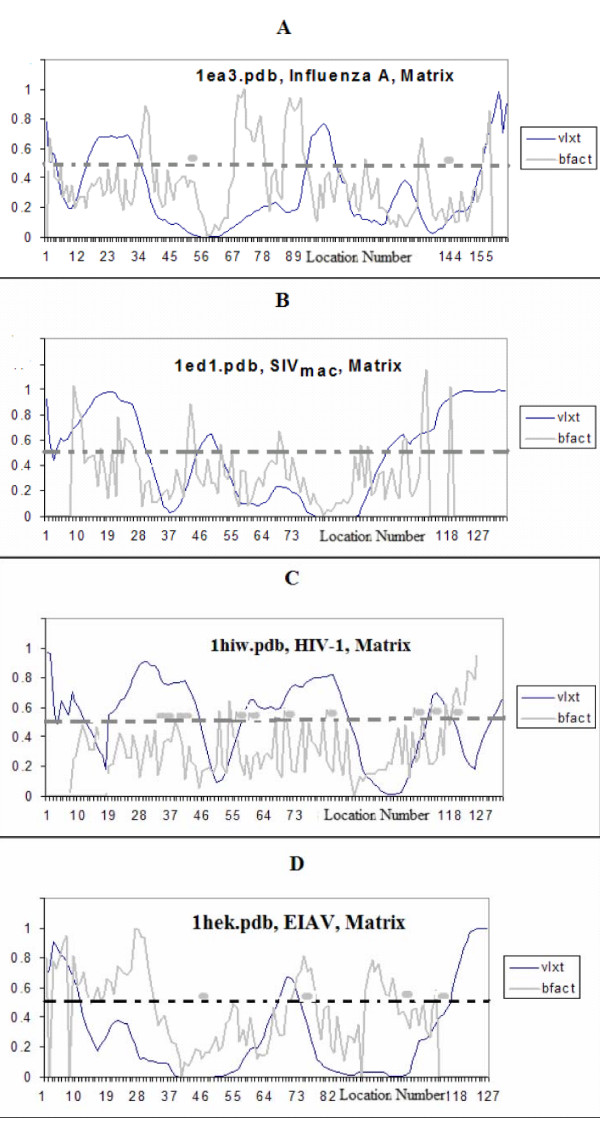
**PONDR/B-factor plots of matrix proteins of various viruses A) the influenza A M1 protein B) the SIV_mac _P17 protein.** C) HIV-1 p17 Matrix protein D) The EIAV p15 Matrix protein. Protein-protein contacts between chains are annotated by thick gray horizontal spots. The normalized B-factor values are seen in the light gray curves. PONDR-VLXT scores are seen in the black curve in each plot.

While Figure 1 and Table 1 represent the predicted disorder of whole polypeptide chains, the PONDR^® ^VLXT plots in Figure 2 represent per-residue distributions of disorder scores. They can be used to measure and compare factors that are not easily quantifiable. For example, Figure 2 allows us to correlate the protein-protein contact sites (when such data are available) with the disorder score profiles. It also compares the normalized B-factor values [42] with the PONDR^® ^VLXT plots.

Analysis of Figure 2 shows that contact sites (shown by thick horizontal gray lines) always correlate either with high B-factors or with high PONDR^® ^VLXT scores suggesting that highly flexible regions of matrix proteins are responsible for protein-protein interactions. For example, contacts between the subunits of HIV-1 p17 are located near or within regions predicted to be disordered, whereas contact sites of the EIAV p15 are mostly located in regions with high B-factor. These observations are in a good agreement with earlier studies which established the usefulness of intrinsic disorder for protein-protein interaction [19-21,23,25,29-31,35,39,40,43-46].

### 3-D Structures with Predicted Disorder

**Figure 3:**
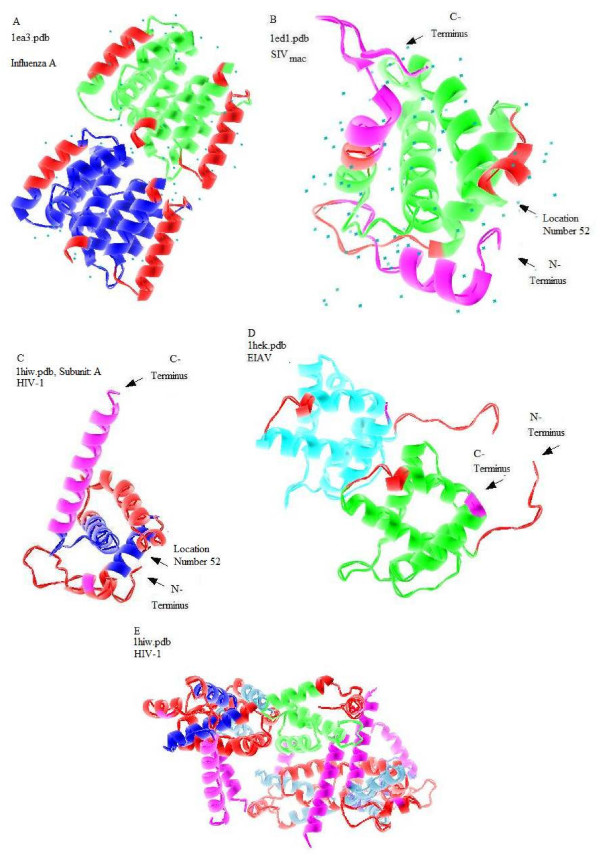
**The three dimensional structures of the matrix proteins of the various viruses with predicted disorder annotation by red and magenta colors.** A) The influenza m1 protein B) SIVmac p17 Protein C) HIV-1 p17 protein shown as a monomer D) The EIAV p15 E) The HIV-1 p17 shown as a multimer. The regions in magenta are regions predicted to be disordered by VL3 (and probably also by VLXT). By contrast, the regions in red are areas predicted to be disordered by VLXT.

Figure 3 provides 3-D representations of the matrix proteins from various viruses. The areas in magenta are the protein regions predicted to be disordered by PONDR^® ^VL3 (and probably PONDR^® ^VLXT also), whereas the regions marked in red are those predicted to be disordered by PONDR^® ^VLXT. Different colors such as yellow and green are used to denote different subunit regions. This presentation of structured proteins allows visualization of regions with the intrinsic propensity for being highly flexible.

## Discussion

### HIV-1 Versus HIV-2 and SIV_mac_: Missing Regions Predicted to be Disordered

#### SIV_mac _is Very Similar to HIV-2

The HIV-2 and HIV-1 viruses, while related, differ in substantial ways in term of immune response, infection, and the onset of AIDS [8,11,12]. SIV_mac _is a subtype of SIV, which was first found in macaques and is known to be very closely related to HIV-2 [10]. While development of AIDS symptoms are seen in virtually all HIV-1 infected patients, AIDS symptoms of HIV-2 infection appears only in a small percentage of patients. We believe that a comparative analysis of PID in related viral proteins could shed some light on the reasons behind these behaviors.

#### PID Rates of Matrix Proteins Correlate with the Difficulties in Finding Vaccines

A brief glance at Table 1 and Figure 1 shows that the PID rates of SIV_mac _and HIV-1 are quite similar, even though the percentage of PID in SIV_mac _p17 (50% by PONDR^® ^VLXT) is smaller than that in HIV-1 p17 (61%). The similarity in the level of PID is likely indicative of the ability of both viruses to evade the immune system. Further support for this hypothesis can be retrieved by analyzing the level of predicted disorder in the influenza M1 protein and in the EIAV p15 protein. Matrix proteins of both of the viruses have low percentage disorder rates, 25% in M1 and 21% in p15. Interestingly, effective vaccines were developed for both of these viruses, even though the mutation rates of the influenza virus is extremely high causing well-known difficulties in the development of new vaccines. Apparently, the PID rate is a good predictor of the ease of vaccine development of a virus. This should not be surprising as our earlier study [41] suggested that the viral matrix likely helps viruses to evade detection by the immune system due to its highly dynamic nature and constant motions. This dynamic behavior is correlated with the high propensity of matrix proteins for intrinsic disorder. Furthermore, it has been hypothesized that the role may be intertwined with the glycoprotein on the surface acting as a broom in a sweeping motion provided by the matrix [41]. This highly dynamic nature of the viral surface may explain the difficulties in the development of vaccine for HIV.

#### Qualitative Differences in Predicted Disorder and Protein-Protein Interactions

Even though the rates of predicted disorder in the SIV_mac _and HIV-1 p17 proteins seem to be similarly high, the PONDR^® ^VLXT plots revealed subtle differences in the disorder distribution within the protein sequences. Figures 2B and 2C show that a long region predicted to be disordered by HIV-1 p17 (53–76 fragment) is missing in SIV_mac _p17. Figures 3B, 3C, and 3E illustrate that this fragment in HIV-1 p17 forms an α-helix and is involved in protein-protein interactions between the subunits. In fact, residues 70–73 from one subunit contact with residues 71, 60, 40, and 46 from another subunit. Analysis of Figure 2C revealed that all these inter-subunit contact sites are located within the PID regions. Therefore, intrinsic disorder plays a crucial role in the inter-subunit interactions, which can be classified as disorder-disorder type of contact. The lack of a predicted to be disordered segment in HIV-2 and SIV_mac _which seems to be crucial for inter-subunit contacts suggests that disorder-disorder protein-protein interactions are replaced by the order-disorder or order-order interactions.

#### Predicted Disorder Patterns Correlate with High B-Factors

Figure 2 shows that, in general, there is a rather good correlation between the predicted disorder patterns and the normalized B-factor curves. For example, the 79–95 fragment of the HIV-1 matrix protein is both predicted to be disordered and is characterized by the high normalized B-factor values (Figure 2C, 1hiw.pdb). In several occasions, there are noticeable lags between the PONDR^® ^VLXT and B-factor curves, as it is seen, e.g. in Figure 2A (M1 matrix proteins of the influenza A virus, 1ea3.pdb), where large B-factor peaks are seen in the 70–90 region, whereas the corresponding PID fragment is located in the 90–105 region.

### HIV *versus *EIAV: Higher Predicted Disorder in HIV

#### Matrix of EIAV Is Relatively Ordered

Matrix protein of EIAV was predicted to be less disordered than that of HIV (see Table 1 and Figure 1). However, even in this case less abundant PID regions could be crucial for the inter-subunit interactions. In fact, analysis of the crystal structure of the p15 protein revealed that residues 46 and 78 of the chain A are involved in interaction with the residues 114 and 105 of the chain B. All these interaction sites are shown as thick gray lines in Figure 2D, which clearly indicates that the interactions between the 15 subunits are less rigorous than that of HIV-1 p17 subunits and can be ascribed to the order-disorder contact type. Since EIAV is from the same genus as HIV, that is, lentiviranae [9,15], these data suggest that the high PID levels are not a common characteristics of the retroviradae family, or even the lentiviranae genus. Apparently, the high level of intrinsic disorder in the matrix proteins is a characteristic feature of HIV-1 and its closest relatives, SIV and HIV-2. These differences in the abundance of disorder seem to be largely constrained to the matrix proteins as the capsids of both HIV-1 and EIAV viruses are quite disordered by prediction (48% and 30% by PONDR^® ^VLXT, see Table 1).

#### Predicted Disorder Patterns of EIAV Are Closer to Those of Influenza than of disorder patterns of HIV/SIV

Analysis of Figure 2 reveals that the pattern of the predicted disorder in EIAV matrix protein is closer to that of the influenza virus than to the disorder profiles of the EIAV's cousins HIV and SIV. Furthermore, EIAV and Influenza A matrix proteins are similar in their relatively low percentages of the predicted disorder (21% in EIAV and 25% in Influenza). The other similarity has to do with the interaction mode between the matrix protein subunits. In fact, contact sites of both Influenza A and EIAV matrix proteins can be classified as disorder-order contacts. In the case of HIV-1, most of the contact sites between the subunits are predicted disorder-disorder interactions. Comparison of the disorder and B-factor profiles of the HIV-1 and SIV_mac _p17 proteins allows extrapolation to be made of the potential modes of inter-subunit interactions in SIV_mac _p17. In fact, if potential interaction sites are distributed similarly within the amino acid sequences of HIV-1 and SIV_mac _p17 proteins, then at least some of the SIV_mac _p17 inter-subunit interactions site can be assigned as disorder-disorder interactions (e.g. if the residue 111 of one SIV_mac _p17 subunit is in contact with the residue 97 from another subunit, then disorder-disorder contact takes place as both of these residues are predicted to be disordered, as seen in Figure 2B).

### High Intrinsic Disorder and Immune Response

#### Potential Implications of More Disordered Matrix Proteins: Immune Evasion

The question then arose: What are the potential implications of more rigid or more disordered matrix proteins? It is likely that more rigid p17 proteins may be less effective in evading immune response. This may be a reason why HIV-2 and SIV_mac _are less pathogenic than HIV-1. It is generally assumed that HIV-2 is less pathogenic than HIV-1 because of the fact that HIV-2 has lesser affinity for CD4 than HIV-1. On the other hand, our data show that there are subtle but important differences between HIV-1 and SIV_mac _(HIV-2) in their patterns of predicted disorder distribution, which also might contribute to the virus's ability to evade the host immune system.

#### Implication to the Search for HIV Vaccines

Our findings might also have some implications to the search for HIV vaccines. One possibility is related to the use animal models and SIV_mac _as in the search for HIV vaccination and drugs. SIV_mac _and SIV_stm _were the first subtypes found in laboratory macaques [13]. Asian primates such as macaques, unlike their African cousins, developed AIDS on the average of 10 years after infection [8]. For this reason, the use of SIV on Asian monkeys has become the standard animal model [47]. However, the extrapolation of data from animal models to HIV in human remains a challenge. Our results suggest that some of these challenges could be explained by the differences in disorder prediction between HIV-1 and SIV (or HIV-2). It is also important to remember that although the high levels of mutation caused difficulties in the development of vaccines against new strains of the influenza, there are effective vaccines against specific strains of the virus. Similarly, there are also effective vaccines available of EIAV. Note, matrix proteins of both influenza virus and EIAV are shown in our study to contain less amount of intrinsic disorder.

#### Joint Role of Glycoproteins and Matrix Disorder

**Figure 4:**
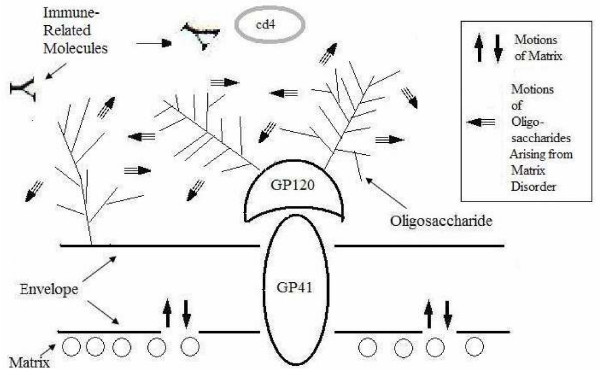
**Schematic diagram: glycoconjugate acts as a broom with sweeping motion arising from matrix.** The striped arrows depict the motions of oligosaccharides arising from the bobbing of the lipid bilayer. The motions of the membrane is also dependent on the matrix for stability or lack of it. The motions of the oligosaccharide may allow and prevent the binding of CD4 and antibodies respectively.

It is established that the HIV envelope glycoprotein gp120 is one of the most glycosylated proteins in nature [48]. Oligosaccharide moieties of viral glycoproteins often hide them from recognition by immune agents such as antibodies [16]. We propose that abnormally disordered matrix proteins might help the surface glycoprotein in eluding immune responses. In other words, intrinsic disorder (read high dynamics) underneath the envelope would work in a tandem with envelope glycoproteins to help viruses in the avoiding of the induction of immune response. The questions then arose: How and why would surface glycoprotein and matrix disorder work in cooperation? A likely scenario is shown in Figure 4. Here, the oligosaccharide moieties of the glycoproteins act as an entropic brush that protects viral surface proteins such as gp120 and gp41 from contacts with immune agents such as antibodies. The matrix protein could then provide the additional motion to the sweep. An advantage of motions that resemble a broom in a sweep is that it enables some regulatory roles via the matrix protein. Earlier it has been already observed that the envelope proteins are very sensitive to the behavior of the matrix proteins [3].

## Conclusion

### Matrix Disorder of Retroviruses Varies with Nature of the Virus

A peculiar finding of this paper is the pattern of predicted disorder of EIAV p15 matching more closely the disorder profile of the influenza M1 protein than those of the matrix proteins of its closer relatives, namely the HIV-1 and SIV_mac _p17 proteins. This feature may be attributed to the ways the viruses are evolved and are transmitted to their hosts. It should be reminded that EIAV is transmitted between horses via insect vectors. In other words, the virus experience dramatic change in the environment during the transmission. It is likely that this mode of transmission has evolutionary requirements similar to those of the influenza virus, which is transmitted via respiratory tract and mucus. HIV and SIV, on the other hand, spread by blood contact or sexual activities. Since it there lesser chance for the exposure to the outside environment in the transmission mode, there is hence lesser evolutionary pressure for the matrix proteins to be ordered. This highlights a role for the matrix protein in many viruses. In many instances, the matrix acts as an encasement for the virion, thereby protecting the virion from damage especially in adverse environments. We have also seen that disorder at the matrix is not an absolute characteristic of retroviruses.

### Implication for the Immune System Invisibility Puzzle of HIV

A single nagging puzzle in the search for vaccines against HIV is the unknown mechanisms helping the virus to evade immune response. Our study suggests that this ability might arise from the abnormal levels of intrinsic disorder at the viral matrix. This hypothesis is supported by the fact that the matrix proteins of other viruses, where vaccines have been more easily found, were predicted to be more ordered. Therefore, there are several ways how disorder predictions can be utilized in the future strategies of the vaccine development. Particularly, one of the new directions in the anti-HIV drug development could be a search for the therapeutic agents able to stabilize the HIV matrix protein.

Another puzzle of HIV viruses is the inability of virologists to account for the waves of the HIV strains seen, even after taking into account the fact that the mutation rate of HIV-1 is 25-times that of influenza. Yet another HIV puzzle is the greater pathogenicity of HIV-1 as compared to HIV-2. It has been generally understood that this is due to the fact that the HIV-1 affinity for CD4 is 28 times greater than that of HIV-2 [49]. Our data suggest that it is not just the affinity for CD4 that give rise to a greater pathogenesis or viral load in HIV-1. Perhaps, it is also the differences in the abilities of the viruses in evading the immune system via disorder at the matrix. This also explains a related observation among epidemiologists that the more easily that HIV-1 spreads sexually the more virulent it becomes [8], since the ease of transmission via blood or sexual intercourse lessens the requirements for a rigid encasement of the virion, which is used in other viruses to prevent virion damage due to harsh environmental factors.

### Potential implications for the Immune Evasion of Cancer Cells and Oncolysis

While this paper has been largely focused on the study of immune evasion as applied to HIV and HIV-related viruses, it may provide a model for immune evasion by other entities, such as cancer cells. There are either very few or no studies done in this area. Perhaps, our results could invigorate interest in this area, given the models and approach used. Furthermore, the results of this paper likely have novel strategic implications for experimental studies on the use of viruses as oncolytic agents, which have often been observed to be rendered ineffective by the immune system. In fact, one of the greatest problems in using the oncolytic viruses is that they are detected by the immune system very quickly so they are only useful for localized treatment of tumors [50]. Our data suggest that this does not have to be always the case and new oncolytic viruses with disordered matrix should be considered.

## Methods

### PDB Accessions

A full description of implementation techniques can be found in a previous paper [41]. The search for important proteins suitable for analysis was done using the Entrez website [51]. Proteins from retroviruses and relatives of HIV were carefully reviewed. The accession codes were grouped into two classes containing proteins whose structures were elucidated using NMR or X-ray diffraction. It should be also noted that suitable data were unavailable for HIV-2. SIV_mac _was used in lieu of HIV-2 since the two are genetically close and the X-ray diffraction data for EIAV matrix and capsid proteins were readily available.

### Query Language

Given the appropriate accessions selected, JAVA programs were used to automatically place the necessary information into the MYSQL database. The data were often checked using the SQL (Sequel Query Language) [52].

### PONDR^® ^VLXT and PONDR^® ^VL3

PONDR^® ^(Predictor Of Natural Disordered Regions) is a set of neural network predictors of disordered regions on the basis of local amino acid composition, flexibility, hydropathy, coordination number and other factors. These predictors classify each residue within a sequence as either ordered or disordered. PONDR^® ^VL-XT integrates three feed forward neural networks: the Variously characterized Long, version 1 (VL1) predictor from Romero *et al*. 2001 [33], which predicts non-terminal residues, and the X-ray characterized N- and C-terminal predictors (XT) from [53], which predicts terminal residues. Output for the VL1 predictor starts and ends 11 amino acids from the termini. The XT predictors output provides predictions up to 14 amino acids from their respective ends. A simple average is taken for the overlapping predictions; and a sliding window of 9 amino acids is used to smooth the prediction values along the length of the sequence. Unsmoothed prediction values from the XT predictors are used for the first and last 4 sequence positions.

PONDR^® ^VL3 combines the predictions of 30 neural networks for the entire protein sequence and was trained using disordered regions from more than 150 proteins characterized by the methods mentioned above plus circular dichroism, limited proteolysis and other physical approaches [36].

### Protein-Protein Contacts and PONDR Plots

In order to detect the locations of protein-protein contacts between the different chains of proteins (i.e., when atoms of neighboring chains are within 3.0 Å from each other), a JAVA program was written to check the interchain atom-atom distance. The program generated graphs with PONDR plots with locations of the protein-protein contacts.

### Three Dimensional Analysis with Disorder Prediction

The JAVA programming language was used to generate codes readable by the molecular 3D software, Jmol [54]. In resulting structures, regions of predicted disorder were annotated by red (VLXT) or magenta (VL3). Areas shaded by magenta were also regions likely predicted to be disordered by VLXT.

### B-Factor

B-Factor values were imported directly from the PDB files into the table access_seq [41] that was modified to accommodate for the B-factor values [42].

## Competing interests

The authors declare that they have no competing interests.

## Authors' contributions

GKMG proposed the idea of the study, carried out the analyses and drafted the manuscript. AKD helped to design experiments and participated in the manuscript drafting. VNU coordinated the studies, participated in their design and helped to draft the manuscript. All authors read and approved the final manuscript.
